# A Case of Essential Thrombocythemia and IgA Nephropathy with Literature Review of the Concurrence

**DOI:** 10.1155/2019/5086963

**Published:** 2019-09-02

**Authors:** Shoja Rahimian, Timothy Johnson, Ronald Herb

**Affiliations:** Department of Internal Medicine, Reading Hospital-Tower Health, USA

## Abstract

Myeloproliferative neoplasms such as essential thrombocythemia (ET) have been associated with glomerular disease on rare instances. A case of ET associated with immunoglobulin A nephropathy (IgAN) is described in a 57-year-old man with a history of hypertension. Progressively worsening renal function was noted in the patient along with unexplained mild thrombocytosis. Pathological review of renal biopsy identified IgAN concurrently with newly diagnosed JAK2-mutated ET. The patient was started on aspirin therapy and closely monitored for his renal function. A literature review of the association of ET and renal disease revealed nine cases of ET associated with IgAN, focal segmental glomerulosclerosis, and fibrillary glomerulonephritis. Comparison of the pathological features of the renal biopsies within the cases noted mesangial proliferation as a common finding, which has been described to be potentiated by platelet-derived growth factor (PDGF). This commonality may represent a link between ET and glomerular disease which deserves further attention in future cases. Improved management of such cases depends on the recognition of the combined occurrence of ET and glomerular diseases and uncovering the shared pathogenesis between platelets and glomeruli.

## 1. Introduction

Essential thrombocythemia (ET) is a type of myeloproliferative neoplasm (MPN) that results in an increased number of platelets in circulation. The current 2016 WHO classification for diagnosis of ET requires major criteria of a platelet count equal to or over 450 × 10^3^/L, demonstration of *JAK2*, *MPL*, or *CALR* mutation, bone marrow biopsy with mature megakaryocyte proliferation but without significant production of neutrophils, erythrocytes, or reticulin fibers, and lack of meeting criteria for other myeloproliferative diseases. ET can alternatively be diagnosed by meeting three of the major criteria in addition to lone minor criteria of a clonal marker or absence of evidence for reactive thrombocytosis [1, 2].

The presence of distinct genotypes is central to both the diagnosis and, at times, treatment of MPNs. Where some MPNs are the result of an individually distinct mutation, others may stem from various genetic aberrations. For instance, polycythemia vera (PV), a form of MPN that leads to an increased concentration of hemoglobin and hematocrit, is associated with a *JAK2V615F* mutation that has been seen in approximately 95% of cases. In contrast, *JAK2* mutations are typically seen in 50-60% of ET cases [3]. In ET cases that lack *JAK2* mutation, *CALR* and *MPL* genes have been shown to possess mutation. Past cohorts studying ET patients found that these mutations (*JAK2*, *CALR*, and *MPL*) are seen in 62-64%, 22-24%, and 4%, respectively [4, 5].

While patients with ET are at a known increased risk for thrombotic conditions such as cerebral vascular accidents, myocardial infarction, pulmonary embolism, and pregnancy complications, there is a lesser known association between ET and glomerulonephropathy. Various forms of glomerulonephropathy have been reported in patients with ET including IgA nephropathy (IgAN), focal segmental glomerulosclerosis (FSGS), diffuse mesangial sclerosis, and fibrillary glomerulonephritis [6–8]. Described here is a case of a 57-year-old man diagnosed with ET that was subsequently diagnosed with IgAN.

## 2. Case Description

A 57-year-old Caucasian man with a history of chronic kidney disease (CKD), essential hypertension, migraines, and obstructive sleep apnea presented to the clinic for establishment as a new patient. Besides occasional migraines, he did not have any other immediate complaints. His blood pressure was mildly elevated at 148/92 mmHg while on lisinopril 10 mg and propranolol 160 mg daily, and physical examination was benign, including lack of lymphadenopathy, rashes, or edema. Review of his laboratory results was notable for worsened renal function in the past year from a serum creatinine (SCr) of 1.10 mg/dL and globular filtration rate (GFR) of 69.25 mL/min to a SCr of 1.66 mg/dL and GFR of 42.91 mL/min. Initially, this was attributed to his angiotensin-converting enzyme inhibitor (ACE-I); however, its discontinuation did not result in recovery of renal function. Additional review of records dating 6 years ago revealed thrombocytosis of around 600 × 10^3^/*μ*L which was more recently in the 418‐440 × 10^3^/*μ*L range. The patient had never experienced arterial or venous thrombotic events, and etiology of his thrombocytosis had not been investigated in the past. Several laboratory studies were ordered with focus on his CKD and thrombocytosis.

Thrombocytosis was pursued with genetic mutation testing, which returned with positive *JAK2* mutation and negative BCR-ABL1. The patient was diagnosed with ET and was started on aspirin. Hydroxyurea therapy was not indicated given a platelet count of 419 × 10^3^/*μ*L.

Two months later, investigation of his progressive chronic kidney disease had revealed proteinuria with 560 mg/24 hours urine protein content, and he was seen by a nephrologist. The electrolytes, uric acid, glucose, serum lipid profile, hepatitis panel, liver function tests, prostate serum antigen, and serum complement levels of C3 and C4 were normal. Serum and urine protein electrophoresis did not identify significant paraproteins. Further testing for autoimmune and systemic disease was negative, which included sedimentation rate, C-reactive protein, antinuclear antibody, and antiproteinase 3. Ultrasonography revealed normal renal parenchymal echogenicity of both kidneys, which measured 10.9 cm in length on the right and 9.9 cm on the left. There was minimal postvoid bladder residual volume of 21 mL, and no hydronephrosis or solid renal mass was seen.

**Figure 1:**
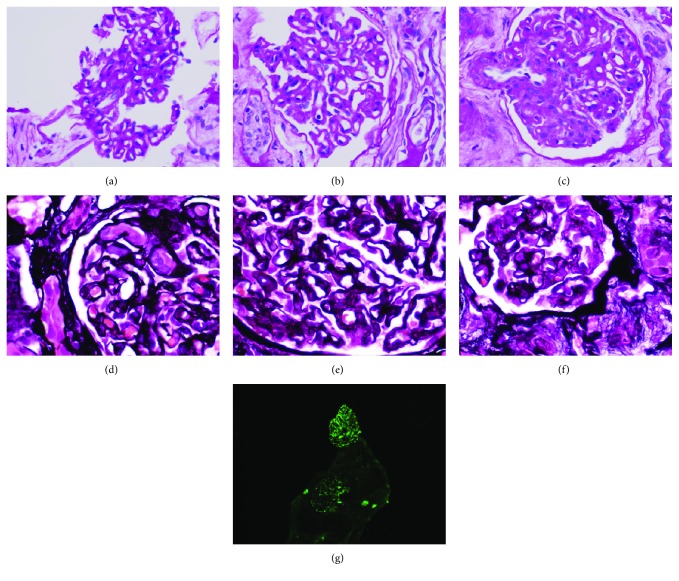
Renal biopsy pathology. Up to fourteen glomeruli were identified under light microscopy, and analysis found glomeruli with diffuse thickening of capillary membranes (a, b). There was mild mesangial matrix expansion and hypercellularity (c, d). There was no evidence of glomerular sclerosis. Segmental duplication of capillary walls with accumulation of eosinophilic deposits was noted between the duplicated membranes. Silver stain reveals thickened capillary walls, duplication of basement membranes with eosinophilic deposits in between (e, f). Trichrome stain showed mild to moderate focal interstitial fibrosis associated with tubular atrophy and drop out with scattered lymphocytes within the fibrotic interstitium. No acute tubulitis was seen. Electron microscopy found diffuse effacement of podocyte foot processes and prominent thickening of capillary loops by a combination of subendothelial deposits, basement membrane duplication, and mesangial cell interposition. On immunofluorescence, there was a diffuse 2 to 3+ mesangial reaction and segmental capillary loop reaction for IgA, IgM, C3, kappa, and lambda (g). The reaction for IgG was only 1+, and there was no reaction for C1q or fibrin. Pronase-retrieval IgG stain on paraffin-embedded tissue was negative. The results indicated a diagnosis of IgA predominant nephropathy.

The patient agreed to undergo a renal biopsy. The specimens were prepared in periodic acid-Schiff, trichrome and silver stains. Microscopic analysis resulted in a diagnosis of IgA predominant nephropathy (Figure 1).

In the following months, the patient was monitored routinely for findings such as hematuria, increased proteinuria, and swelling. He was continued on lisinopril for mild proteinuria. Repeat testing 3 months after his prior results found an elevated SCr of 1.91 mg/dL and GFR of 36.50 mL/min. Urine testing at that time however found improved proteinuria with 23 mg/24 urine. On subsequent follow-up visits, the patient's blood pressure remained in the range of 118-140/82-92 mmHg. His platelet count in the 6 months after his renal biopsy remained consistently between 797 and 876 × 10^3^/*μ*L without cytoreductive therapy. Subsequently, given that his SCr remained 1.6-1.7 mg/dL with proteinuria less than 300 mg per day, it was decided to continue the ACE-I without immunosuppressive therapy unless further progression became evident.

## 3. Discussion

The development of glomerulonephropathy in patients with MPNs is not well understood and is infrequently encountered. While cases have been described within many of the myeloproliferative neoplasm subtypes, this association seems to be more commonly documented within PV and primary myelofibrosis (PMF). Cases of PV describing the development of nephrotic range proteinuria often report FSGS following renal biopsy [7–9]. While FSGS is described in most PV cases, some cases of PV-related nephropathies have also described IgA nephropathy [10]. Furthermore, there is a reported case for which Said et al. proposed the term “MPN-related glomerulonephropathy” to distinguish MPN-related glomerular disease from other common glomerular diseases based on histopathologic findings. MPN-related glomerulonephropathy was characterized by (1) mesangial sclerosis and more pronounced mesangial proliferation, (2) a lack of nodular mesangial sclerosis, (3) absence of immune deposits, (4) presence of intracapillary hematopoietic cell(s), and (5) segmental duplication of the glomerular basement membrane with findings that mimic chronic thrombotic microangiopathy (TMA) but without intracapillary fibrin thrombi, arteriolar thrombotic lesions, or features of microangiopathic hemolytic anemia [11].

Similar to PV, cases that describe ET-related glomerulonephropathy often report findings of FSGS (Table 1). Nine other cases were identified of glomerulonephropathy in patients with ET and seemingly no other etiologic explanation in a PubMed database search of the English literature [12–15]. Our case presented above marks the tenth ET-related nephropathy case and the second describing IgAN in a patient with ET.

On comparison of the ET and glomerulonephropathy previously described cases with ours, only the ET case in Said et al. had the histological appearance of the proposed MPN-related glomerulonephropathy. Four of the ten were consistent with FSGS, two were not given an official diagnosis, two IgAN (including our case), one fibrillary glomerulonephritis, and one MPN-related glomerulonephropathy. Mesangial proliferation was a common finding as it is stated in 9 out of 10 of the cases. Another is immune complex deposition, being stated in 6 out of the 10. Our case shared the mesangial proliferation and deposition of immune complexes, which may be an affected process with the shared pathophysiology of ET.

A possible pathogenic correlation between ET and glomerular disease could be with platelet-derived growth factor (PDGF) and its role in fibrotic processes such as glomerulosclerosis and myelofibrosis. In a study of fibrogenic growth factors in IgAN and FSGS, Stein-Oakley et al. concluded that these glomeruli express higher levels of PDGF receptors which were strongly associated with disease severity, particularly with FSGS. Likewise, the amount of mesangial proliferation was associated with the higher expression of PDGF in glomeruli of patients with IgAN and FSGS [16]. In studies comparing ET and PDGF levels, mutations of *JAK2*, *CALR*, and *MPL* have been linked to increased concentrations of PDGF, with *CALR* mutations leading to three times higher levels than with the other mutations. This has been used to offer an explanation as to the significantly higher incidence of primary myelofibrosis in patients of ET and *CALR* mutation [17].

Increased fibrogenicity as a result of ET mutations also stems from transforming growth factor-*β* (TGF-*β*). TGF-*β* has also been proposed to have a role in glomerular diseases in a process of podocyte injury that is described in the early stages of FSGS. In the FSGS and ET case described by Haraguchi et al., higher levels of TGF-*β* as well as PDGF were found [14].

Similarly, PV-associated nephropathies have been thought to involve fibrogenic cytokines such as PDGF. Additionally, it has been proposed that with the resultant hyperviscosity of PV, chronic increases in blood volume and viscosity can lead to vascular damage to the intima of vessels, with subsequent microthrombi causing renal capillary occlusion that ultimately decrease glomerular filtration. This is supported with findings of pronounced hypertension and hyperuricemia in patients with PV-related glomerulonephropathy (8 of 23 described cases being IgA nephropathy) [10].

In the present case, cytoreductive therapy was not found to be necessary given the lower risk and stable levels of thrombocytosis. Interestingly, the use of anagrelide in ET has been found to decrease the levels of PDGF which may carry an important implication in the treatment of glomerulonephropathies in patients with ET [18]. In consideration of the importance of PDGF in the shared pathogenesis of these conditions, other cytoreductive therapies such as interferon and hydroxyurea would benefit from a study of their effects on plasma PDGF levels. Even so, potential renal impairment directly from these agents should be considered if treatment is initiated. MPN patients who are on cytoreductive therapies with profound renal impairment, which does not recover with dosage decrease, may benefit from renal biopsies to identify a concurrent glomerulonephropathy.

In the case described above, *JAK2* mutation was present and the glomerular injury had shown mesangial proliferation. Glomerular sclerosis was not present, which may depend on the specific mutation of ET and the subsequently induced level of fibrogenesis. The other cases did not all specify which ET gene mutation was found. This could be an area of improvement as the role of ET and fibrogenesis in glomerulonephropathy is further investigated.

## 4. Conclusion

As it stands, the rare combination in the present case of ET and IgAN will likely proceed with separate management of the renal disease and the ET. Until a clear understanding of the combined pathogenesis is established, platelet numbers will dictate only the treatment of ET and not worsening of the renal function. Practice-changing management strategies can be obtained if clinicians can recognize the concurrence of ET and glomerulonephropathy, and future studies are developed to better clarify associations of ET mutations and renal histopathological findings.

## Figures and Tables

**Table 1:** Biopsy results of essential thrombocythemia and glomerulonephropathy cases.

Case	Sex	Age	Renal biopsy findings	Renal diagnosis	Time after ET diagnosis	Source
1	Male	68	Mesangial PAS (+) fibrillary deposits without mesangial proliferation or crescent formation (+) IgG on immunohistochemical staining Weakly (+) IgA, IgM, and C3	Fibrillary glomerulonephritis	30 years	Asaba et al. [6]
2	Male	25	Diffuse mesangial sclerosis with proliferation Segmental capillary thickening immunohistochemical staining (-)	FSGS	Unknown	Au et al. [7]
3	Female	39	Global & segmental sclerosis Segmental mesangial IgM and C3 deposits	FSGS	Unknown	Au et al. [7]
4	Female	70	Mesangial proliferation Foot process effacement on electron microscopy Mesangial deposits (-) for immunoglobulin or complement	N/A	24 years	Usui et al. [8]
5	Female	74	Glomerular sclerosis Diffuse mesangial proliferation Features of chronic thrombotic microangiopathy Glomerular basement membrane thickening	Myeloproliferative neoplasm-related glomerulopathy	7 years	Said et al. [11]
6	Female	63	Mesangial proliferation IgM (+) on immunohistochemical staining Weakly IgA (+) on immunohistochemical staining Without mesangial deposits on electron microscopy	N/A	3 years	Fujita and Hatta [13]
7	Female	76	Mesangial proliferation with crescent formation IgG and IgA (+) on immunohistochemical staining Dense deposits in the mesangium on electron microscopy	IgA nephropathy	3 years	Fujita and Hatta [13]
8	Male	76	Segmental sclerosis and hyalinosis Segmental IgM (+) deposits Foot process effacement on electron microscopy	FSGS	0 (same time diagnoses)	Haraguchi et al. [14]
9	Male	75	Glomerular sclerosis Glomerular basement membrane thickening with mesangial proliferation	FSGS	4 years	Saigusa et al. [15]
10	Male	59	Mesangial hypercellularity Membrane thickening with mesangial proliferation IgG and IgA (+) on immunohistochemical staining Foot podocyte effacement and subendothelial on electron microscopy	IgA nephropathy	2 months	Rahimian (2019)
